# Factors Determining the Susceptibility of Bacteria to Antibacterial Photodynamic Inactivation

**DOI:** 10.3389/fmed.2021.642609

**Published:** 2021-05-12

**Authors:** Aleksandra Rapacka-Zdończyk, Agata Woźniak, Klaudia Michalska, Michał Pierański, Patrycja Ogonowska, Mariusz Grinholc, Joanna Nakonieczna

**Affiliations:** ^1^Department of Pharmaceutical Microbiology, Faculty of Pharmacy Medical University of Gdańsk, Gdańsk, Poland; ^2^Laboratory of Molecular Diagnostics, Intercollegiate Faculty of Biotechnology, University of Gdańsk and Medical University of Gdańsk, Gdańsk, Poland

**Keywords:** membrane fluidity, staphyloxanthin, antioxidant enzymes, transcription regulators, reactive oxygen species, DNA photodamage

## Abstract

Photodynamic inactivation of microorganisms (aPDI) is an excellent method to destroy antibiotic-resistant microbial isolates. The use of an exogenous photosensitizer or irradiation of microbial cells already equipped with endogenous photosensitizers makes aPDI a convenient tool for treating the infections whenever technical light delivery is possible. Currently, aPDI research carried out on a vast repertoire of depending on the photosensitizer used, the target microorganism, and the light delivery system shows efficacy mostly on *in vitro* models. The search for mechanisms underlying different responses to photodynamic inactivation of microorganisms is an essential issue in aPDI because one niche (e.g., infection site in a human body) may have bacterial subpopulations that will exhibit different susceptibility. Rapidly growing bacteria are probably more susceptible to aPDI than persister cells. Some subpopulations can produce more antioxidant enzymes or have better performance due to efficient efflux pumps. The ultimate goal was and still is to identify and characterize molecular features that drive the efficacy of antimicrobial photodynamic inactivation. To this end, we examined several genetic and biochemical characteristics, including the presence of individual genetic elements, protein activity, cell membrane content and its physical properties, the localization of the photosensitizer, with the result that some of them are important and others do not appear to play a crucial role in the process of aPDI. In the review, we would like to provide an overview of the factors studied so far in our group and others that contributed to the aPDI process at the cellular level. We want to challenge the question, is there a general pattern of molecular characterization of aPDI effectiveness? Or is it more likely that a photosensitizer-specific pattern of molecular characteristics of aPDI efficacy will occur?

## Introduction

Antibacterial photodynamic inactivation (aPDI) is a therapeutic option used in the treatment of infectious diseases. It is based on a combination of a photosensitizer (PS), light and oxygen to remove highly metabolically active cells. These cells may be microorganisms, such as fungi (1), viruses (2), or bacteria (3). The main element in aPDI is a triplet excited photosensitizer, whose action can lead to the formation of singlet oxygen and radicals, known as reactive oxygen species (ROS) (4). These reactive species cause damage to biological molecules, which promotes cell death, the desired effect of aPDI.

In comparison with other methods of treatment, aPDI has several advantages. Photoactivation allows local treatment, which reduces the side effects of photodynamic therapy. In addition, aPDI has several cellular targets, and therefore, it is not considered to lead to the development of resistance to the treatment. However, despite these advantages and increasing knowledge about the effectiveness of photodynamic inactivation, the clinical application of aPDI is still not widespread and mainly concerns anticancer applications (5). Thus, researching the molecular mechanisms involved in the photoinactivation of microorganisms warrants serious attention. Many researchers have focused exclusively on finding new photosensitizers or modifying existing ones to maximize the quantum yield of singlet oxygen in *in vitro* tests, while ignoring molecular aspects within microbial cells and the factors that affect differences in the susceptibility of microbes to photoinactivation. Notably, the essential biological targets in photodynamic reactions for achieving effective eradication of microorganisms are still unclear, as are the genetic or phenotypic features of microorganisms that determine their response to photoinactivation (6). In our opinion, understanding the mechanism and phenomena occurring in microbial cells in response to photodynamic reactions is indispensable for increasing their effective clinical use. The primary motivation for writing this review was to identify the molecular phenomena occurring in the cells of microorganisms during and after photosensitization that may influence the effectiveness of photodynamic inactivation and to identify the elements that determine the existence of microbial phenotypes with differences in vulnerability to aPDI.

Photodynamic inactivation includes aPDI employing exogenously administered photosensitizers (PSs) and antimicrobial blue light (aBL), leading to the excitation of endogenously produced photosensitizing compounds, i.e., intracellular porphyrins and flavins.

**Figure 1:**
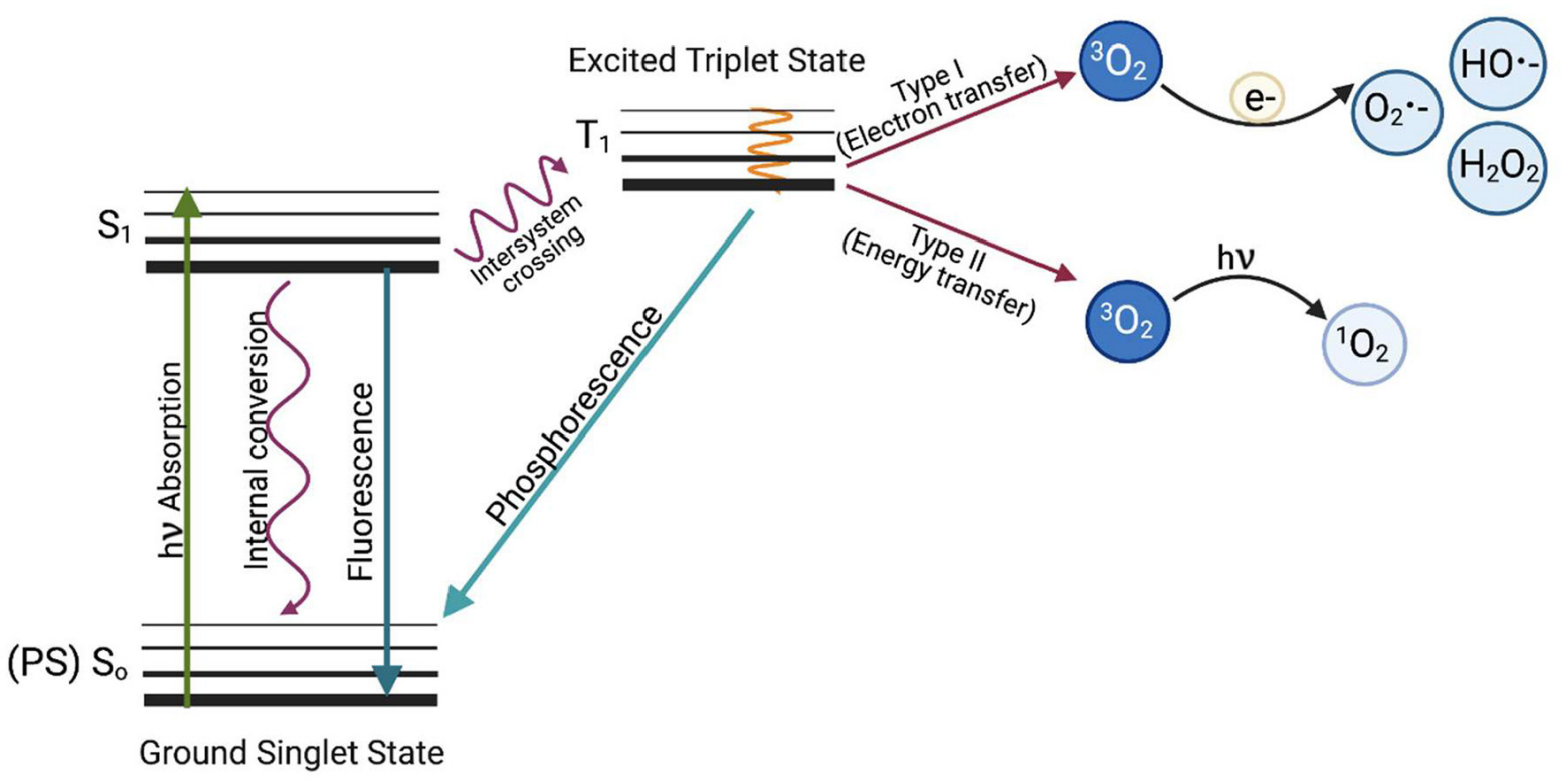
The Jabłoński diagram illustrates the mechanism of photodynamic inactivation. aPDI can be divided into two types of reactions. The type I (electron transfer) reaction occurs when a photon is absorbed by the PS, which causes PS excitation and transition from the ground PS singlet state (So) to the excited PS singlet state (S_1_) and later to the excited triplet state via the inter-system crossing (T_1_). When excited, the photosensitizer can transfer an electron (e^−^) to molecular oxygen (^3^O_2_) to produce oxygen radicals and other reactive oxygen species such as superoxide anion (O_2_^•−^), hydroxyl radical (HO^•−^) or hydrogen peroxide (H_2_O_2_) or can react with biomolecules such as membrane lipids. The first stage of the Type II reaction (energy transfer) is similar, but the photosensitizer in the triplet excited state (T_1_) transfers energy directly to molecular oxygen. This produces highly reactive singlet oxygen (^1^O_2_). Based on Oruba and Chomyszyn-Gajewska (9).

There are two types of reactions that occur in aPDI (Figure 1); a type I reaction, which generates ROS such as superoxide (^·^${\text{O}}_{2}^{-}$), hydroxyl radical (^·^OH), and hydrogen peroxide (H_2_O_2_), or a type II reaction, in which mainly singlet oxygen (^1^${\text{O}}_{2}^{-}$) is produced. Type I and type II reaction products can be produced simultaneously in PDI, with the proportion of each being dependent on the type of photosensitizer used and the ionic strength of its solvent (7). Recently, a type III reaction has been added to the well-known mechanisms of type I and II, in which, regardless of the presence of oxygen, free radicals of inorganic compounds are formed, which can also be involved in the photoinactivation of microorganisms (8).

**Figure 2:**
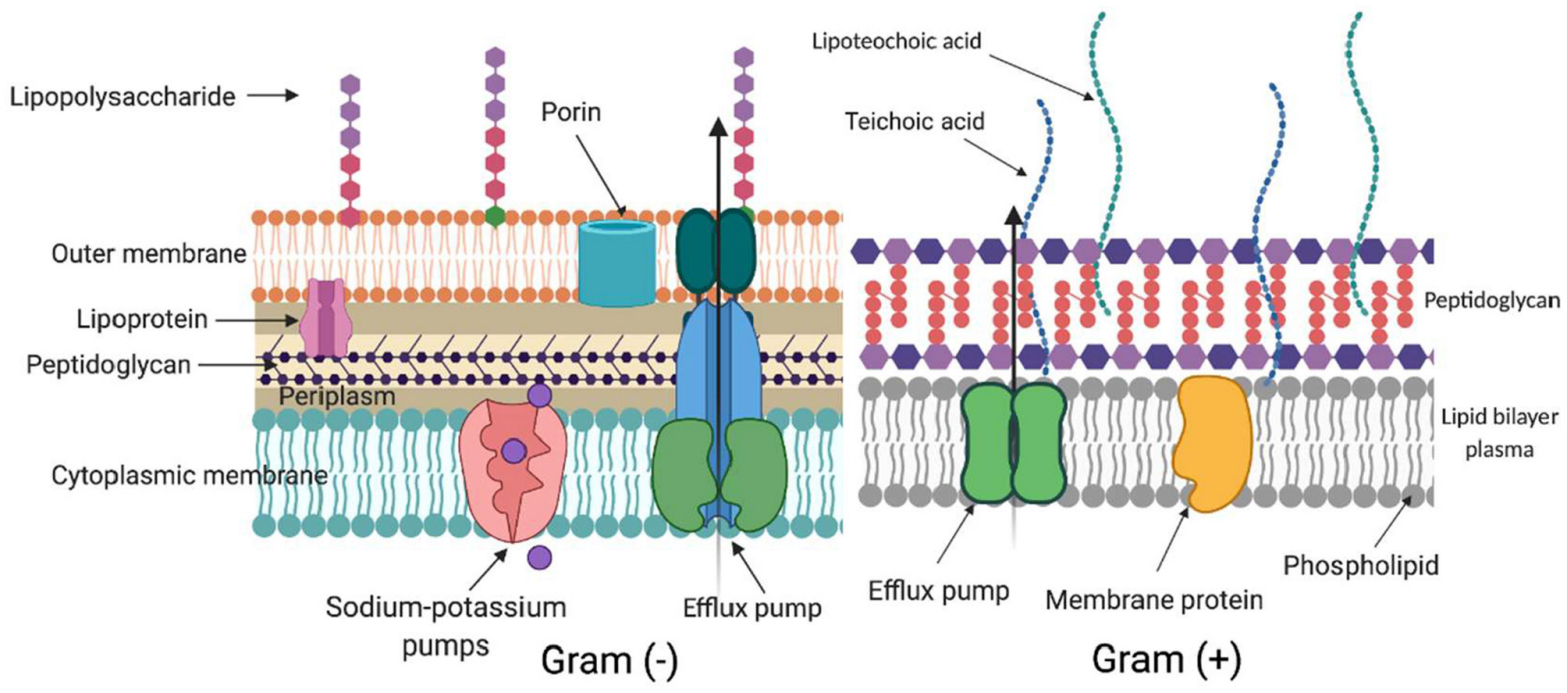
Differences in cell wall structure in gram-negative and gram-positive bacteria. Schematic representation of a cell wall in gram-negative [Gram (–)] and gram-positive [Gram (+)] species. The complex structure of two lipid membranes: cytoplasmic and an outer membrane are responsible for generally lower aPDI effectiveness against gram-negative bacteria. On the contrary, gram-positive species with their thick although relatively porous peptidoglycan are more easily photoinactivated. The main elements in the cell wall that can be involved in photodynamic inactivation are depicted. (Created with BioRender).

**Figure 3:**
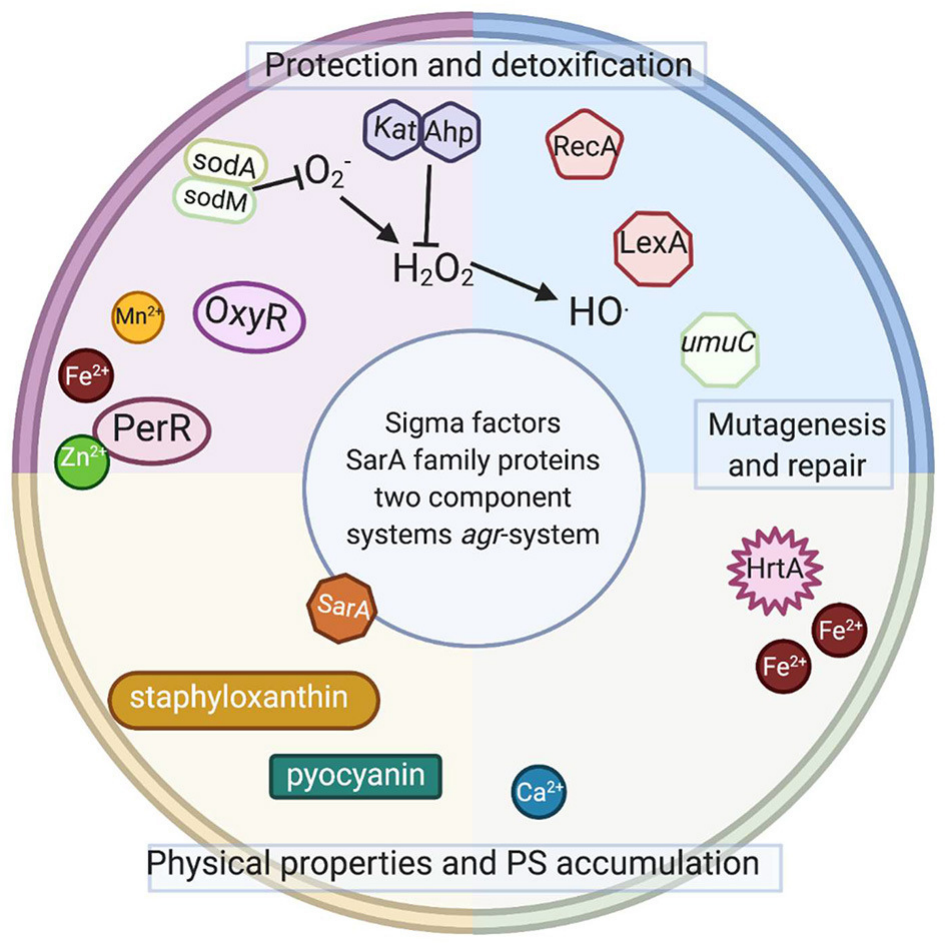
Intracellular elements involved or potentially involved in photodynamic inactivation. Schematic representation of intracellular elements that were studied with respect of photodynamic inactivation of bacteria. Sod, superoxide dismutase; OxyR, PerR, peroxide-responsive regulators in gram-positive and gram-negative species; Kat, catalase in gram-positive and gram-negative species; Ahp, alkyl hydroperoxide reductase in gram-positive and gram-negative species; RecA, recombinase A; LexA, SOS system repressor, *umuC*, gene coding for an error-prone polymerase in gram-positive and gram-negative species; HrtA, heme-regulated transporter A in *S. aureus*; SarA, transcriptional regulator in *S. aureus*; *agr*, virulence accessory gene regulator in *S. aureus*. Staphyloxanthin (membrane embedded) and pyocyanin (secreted) are dyes produced by *S. aureus* and *P. aeruginosa*. Please see the text for detailed explanations (Created with BioRender).

ROS generated during aPDI and aBL target several biomolecules within a cell (Figure 2): membrane lipids, proteins, nucleic acids, carbohydrates, and thiols. Inside a cell (Figure 3), proteins seem to be the main target, as they are the most abundant. Singlet oxygen, which is produced by most porphyrin derivatives, reacts with aromatic amino acids as well as those containing sulfur, which results in the accumulation of toxic products (10). In nucleic acids, the formation of 8-oxo,7,8-dihydro-2'-deoxyguanosine (8-oxo-G) may lead to frameshift mutations (11). ROS cause lipid peroxidation, disturbing the cell membrane integrity and energy production and transport processes (12). Cell envelopes are considered significant photoinactivation targets, which may be supported by the fact that, in general, gram-positive [G (+)] bacteria display higher susceptibility to photoinactivation than gram-negative [G (–)] bacteria. This probably results from differences in the structure of cell envelopes between these two microbial groups (Figure 2). G (+) and G (–) bacteria have a similar cytoplasmic membrane, however, they differ significantly in peptidoglycan thickness and the presence of the outer membrane. The outer membrane in G (–) species constitutes an additional block of the physical interaction of PS with intracellular ROS-sensitive molecules. The thick but quite porous structure of the peptidoglycan in G (+) species filled with teichoic and teichuronic acids is not a very difficult barrier for PS to overcome. The situation is different in G (–) where the outer membrane containing lipopolysaccharides is tight and difficult to penetrate (Figure 2).

It should be remembered that in the case of bacterial cells it is extremely difficult to analyze individual structural elements of the cell, for example by flow cytometry techniques, where the bacterial cells are often at the limit of the resolution of the method. Obtaining information on the role or behavior of individual cell structures in the process of photoinactivation of bacteria requires the use of sophisticated techniques to which access is often limited, such as atomic force microscopy. Gram-negative species have a more complex cell envelope structure, which may explain their higher tolerance to photoinactivation (13). Despite the crucial role of the cell envelope in the bactericidal activity of photoinactivation, the actual contribution of DNA damage to the outcome of phototreatment should not be underestimated.

## Phototreatment Tolerance

Due to their non-selective, multitargeting and ROS-dependent mechanisms of action, aPDI and aBL are considered unlikely to induce bacterial resistance and/or tolerance. Resistance/tolerance development has been extensively studied for both phototreatments (aPDI and aBL) over the past decade (14–29). Kashef and Hamblin reviewed many of these works in depth (30). However, the phenomenon of resistance/tolerance was not observed in any of the publications included in this review (30). Guffey et al. suggested that *S. aureus* may be capable of developing adaptation to blue light irradiation. Subsequent applications of blue light (405 nm) to subcultured generations of *S. aureus* (ATCC 25923) were increasingly useful through four cycles; however, beginning with the 5th cycle, the effectiveness of phototreatment decreased (24). In turn, in studies performed by Amin et al., *Pseudomonas aeruginosa* exhibited reduced susceptibility to sublethal aBL treatment after nine cycles of photoinactivation. The fraction of the surviving cells was increased by approximately two log_10_ units compared to the first cycle (28). Although the authors did not consider these results to reflect resistance, in our opinion, this observation could indicate possible tolerance development. Our studies indicated the development of *S. aureus* tolerance to RB-mediated aPDI and aBL (405 nm) when the *S. aureus* USA300 JE2 strain was subjected to 15 cycles of both sublethal treatments. Potential reductions in susceptibility to aPDI and aBL were examined after the 5th, 10^th^, and 15th consecutive cycles. The developed adaptation was stable after five cycles of subculturing without aPDI/aBL exposure. The development of aPDI/aBL tolerance was also observed in clinical MRSA and MSSA strains as well as in other representatives of gram-positive species, i.e., *Enterococcus faecium* and *Streptococcus agalactiae*. A key point of the obtained results was the lack of cross-tolerance between RB-aPDI and aPDI mediated by other PSs, i.e., [7-(dimethylamino)phenothiazin-3-ylidene]-dimethylazanium chloride (New Methylene Blue, NMB) and 5,10,15,20-tetrakis(1-methyl-4-pyridinio)porphyrin tetra(p-toluenesulfonate (TMPyP), along with a lack of cross-tolerance between aPDI and aBL. It should be highlighted that the developed aPDI/aBL tolerance cannot be considered resistance since the administration of more rigorous conditions, i.e., increased PS concentrations and higher light doses, caused bacterial eradication (20). In our most recent study, we also demonstrated the development of aPDI tolerance (21). The application of 10 cycles of sublethal aPDI resulted in significant tolerance development for all tested *S. agalactiae* strains, including both the reference ATCC 27956 strain and clinical isolates (2306/06 and 2974/07). The developed tolerance decreased aPDI efficacy by up to 3 log_10_ units in viable counts. Moreover, we observed the phenotypic stability of the developed tolerance after passaging the cells for the next five cycles with no selection pressure. The obtained results indicated increased tolerance after passaging that reduced the aPDI efficacy by 5 log_10_ units (21).

The studies mentioned above indicate that the developed adaptation results from genetic alterations and is sustained in subsequent generations without selective pressure. Currently, no data indicate whether the observed tolerance has any particular pattern, i.e., which particular genetic elements underlie the observed phenomenon of increased tolerance to the studied aPDI or aBL applications. Several elements (Figure 3) discussed below might be expected to be involved in the response to the phototreatments.

## Oxidative Stress Sensing and Detoxifying Mechanisms

Since bacterial cells are exposed to ROS produced both internally (aerobic growth, endogenous PSs, e.g., porphyrins) and externally (oxidative burst of macrophages, drugs, environment), there are several elements involved in oxidative stress sensing and ROS detoxification. Most of them are present in both gram-negative and gram-positive bacteria that live in aerobic conditions.

### The General View on Oxidative Stress Sensing and Detoxifying Mechanisms in Gram-Positive and Gram-Negative Bacteria

The main transcription factor involved in sensing oxidative stress in bacterial cells sensing oxidative stress is OxyR. It can detect an increased level of peroxide by thiol-disulfide exchange. Its analog, PerR, acts by a metal-mediated peroxide-sensing mechanism. PerR, as a metalloprotein, contains Zn^2+^ and a second metal ion—Fe^2+^ or Mn^2+^. These proteins have a regulatory function; PerR containing Fe^2+^ responds to low levels of H_2_O_2_, and PerR containing Mn^2+^ responds poorly to elevated H_2_O_2_ levels (32). Furthermore, PerR is one of the ferric uptake regulator (Fur) homologs. PerR is a manganese-dependent transcriptional repressor that controls the transcription of catalase (Kat) and alkyl hydroperoxide reductase (Ahp). In *S. aureus*, PerR can repress Fur transcription and act as an autoregulator. It is induced by elevated iron and H_2_O_2_ concentrations (33). To avoid ROS intoxication due to the Fenton reaction, strict regulation of the free iron concentration is necessary. Fur, active as a dimer, binds structural Zn^2+^ and regulatory Fe^2+^. Fur can actively repress transcription by blocking the polymerase binding site of the promoter or control target gene expression via secondary regulators. Fur also positively regulates the expression of iron co-factored superoxide dismutase (*sodM*) and downregulates Mn-containing SodA (superoxide dismutase A). Fur transcription is activated by OxyR, which also activates the transcription of KatG and AhpCF in *E. coli* (34). Another important element activated by OxyR is Mnt, a metal ion-activated transcriptional repressor of manganese transporter genes. Manganese ions are actively imported by MntH mostly after OxyR activation; they are crucial for the activation of SodA and can also substitute iron in some metalloenzymes (35). Furthermore, PerR and OxyR can regulate *mnt* genes in different species. Therefore, Fur, PerR, and MntR form an integrated network controlling peroxide stress resistance as well as Fe^2+^ and Mn^2+^ homeostasis (33). In addition to iron and manganese, zinc, and copper also play important roles in oxidative stress. All of these metals can be cofactors for superoxide dismutases: iron for SodB, manganese for SodA and SodM, and copper/zinc for SodC (36). The main mechanism used by *E. coli* to sense superoxide anions functions via SoxRS. SoxR activates the transcription of SoxS, which in turn activates the production of several enzymes, including superoxide dismutase and DNA repair enzymes (37). Interestingly, the SoxRS system was shown to partially protect cells from singlet oxygen after treatment with (dipotassium;2,3,4,5-tetrachloro-6-(2,4,5,7-tetraiodo-3-oxido-6-oxoxanthen-9-yl)benzoate (rose bengal, RB) and illumination (38).

### Oxidative Stress Elements Studied With Respect to aPDI

During aPDI, the excited state PS can generate ROS, including the superoxide anion (O_2_·-) and, in the presence of divalent ions (Fe^2+^), which enable the Fenton reaction, hydroxyl radical (HO·) or hydrogen peroxide (H_2_O_2_) (type I mechanism). It can also produce highly reactive singlet oxygen, ^1^O_2_ (type II mechanism). Superoxide dismutases and catalases can catalyze the reaction of O_2_·-, HO· and H_2_O_2_ to H_2_O and molecular oxygen, which can allow bacteria to avoid eradication when treated with PSs acting based on type I mechanism. On the other hand, there is no known enzyme specific for the detoxification of singlet oxygen, which makes mechanism type II PSs more promising as antibacterial agents (39).

One of the singlet oxygen producers, [7-(dimethylamino)phenothiazin-3-ylidene]-dimethylazanium chloride (methylene blue, MB), was used to study the *E. coli* response to oxidative damage. Kim et al. observed that the *oxyR* overexpression mutant was much more resistant to singlet oxygen-mediated cellular damage than the *oxyR* deletion mutant (28). The *oxyR* overexpression mutant also exhibited significantly higher activities of antioxidant enzymes such as Sod and Kat, which suggested that the *oxyR* regulon plays an important protective role in singlet oxygen-mediated cellular damage (40). Among many oxidative stress-induced compounds, OxyR can partially control the production of pyocyanin. Hendiani et al. observed pyocyanin gene upregulation after MB photoinactivation (41). Another transcription regulator, Fur, was investigated in an *H. pylori* mutant in which the *fur* gene was replaced with a kanamycin resistance marker. After MB-aPDI, the *H. pylori* mutant showed a 10,000-fold decreased viable cell number compared with wild-type *H. pylori*, which indicates a role of Fur in the response to aPDI-induced oxidative stress. Interestingly, a 3-fold higher increase in the level of 8-oxo-G indicated DNA damage (42).

The high importance of SodA and SodB in the survival of aPDI by *E. coli* was observed by Herbig et al. (32). A mutant that was not able to produce SodA and SodB was more susceptible to MB-PDI than the wild-type strain. However, the addition of external quenching agents such as Sod and Kat was sufficient to protect both strains from MB-aPDI. The addition of these enzymes was not sufficient when both strains were treated with TMPyP-aPDI. In addition, with TMPyP, the differences in survival between strains were not significant. Researchers did not observe changes in the expression of *sodA* and *sodB* for any of the treatments (43). On the other hand, in earlier studies, superoxide dismutase activation was observed after 6 h of growth in the presence of manganese and azure C, MB, thionine, or (7-amino-8-methylphenothiazin-3-ylidene)-dimethylammonium chloride (toluidine blue O, TBO) under room light (33). Additionally, *E. coli B*, with induced levels of superoxide dismutase and catalase, exhibited marked resistance to azure C phototoxicity (44). Misba and Khan performed the analysis of gene expression in *Streptococcus mutans* biofilms after azure A or NMB aPDI (34). They observed a 2-fold increase in expression of the *sod* gene (45). Buchovec et al. also studied the gene expression of *oxyR, fur, sodA, ahpC, ahpF, katG, katE*, and *sodC* using chlorophyllin-based photosensitization against *Salmonella enterica*. They observed significantly increased expression of *ahpC* and a slight increase in *oxyR*. The rest of the genes had expression changes <1.5-fold (46). On the other hand, when Dosselli et al. performed proteomic analysis of *S. aureus* after T4 porphyrin-aPDI, they observed that AhpC showed the most pronounced decrease in the intensity level of proteins and an 8-fold increase in catalase (KatA) protein levels (47). KatA was also investigated in *P. aeruginosa* after treatment with blue light or TBO-aPDI. The isogenic mutant *katA*^−^ was significantly more sensitive to both treatments than the wild-type strain (48). The activity of Sod and Kat was investigated in *E. coli* after treatment with illuminated riboflavin. The activity of both enzymes was significantly reduced in comparison to treatment with light or riboflavin only (49). The activity of Sod was also checked after visible light illumination of *S. aureus* and *E. coli* in the presence of TA/Fe3^+^/AgNP nanofilm (38). The illumination of bacteria in the presence of the nanofilm caused a decrease in the activity of Sod in both species (50). Finally, our research team investigated *S. aureus* Sod isogenic mutants deprived of either *sodA* or *sodM* or both genes. We did not observe differences in the effectiveness of protoporphyrin IX (PPIX)-aPDI against all strains tested. However, in the medium without Mn^2+^ ions, the double *sodAM* mutant was highly susceptible to aPDI (39). We also examined 8 clinical isolates of *S. aureus* (4 MRSA and 4 MSSA), including strains that were highly resistant and strains that were highly vulnerable to photodynamic inactivation. We observed that Sod activity as well as *sodA* and *sodM* transcript levels increased after PPIX-based aPDI but only in aPDI-sensitive strains. The fact that an increase in Sod activity is observed only in aPDI-susceptible cells emphasizes that this is probably not a direct factor affecting *S. aureus* vulnerability to PPIX-based aPDI (51).

On the other hand, in our recent research concerning *S. agalactiae* tolerance to RB-aPDI, we observed that immediately after sublethal treatment, *sod* expression increased in both the control and tolerant strains, while *ahpC* expression decreased in both (21).

In conclusion, few studies have investigated the role of oxidative stress sensing and detoxifying mechanisms in photoinactivation. Different factors were examined in different bacterial species and after treatment with different photosensitizing compounds. We still lack profound knowledge of the aPDI mechanism of action and therefore possible bacterial actions that allow them to escape applied treatment. Does the complex regulatory antioxidant system in bacteria confer protection against aPDI? The answer seems to be positive, as many studies indicate that antioxidant enzymes are necessary to protect microbial cells from aPDI-induced ROS. Another issue remains unsolved, however: whether aPDI-generated ROS can specifically induce the production of enzymatic detoxifying enzymes, which might further lead to adaptation to elevated levels of ROS.

## Key Master Regulator of Stress Response

### Agr

One of the most important and best-studied regulatory systems in *S. aureus* is the accessory gene regulator (Agr). Agr was first described in 1986 and plays a crucial role in the pathogenesis of *S. aureus*, responding to various signals from the environment—availability of energy, bacterial cell density (quorum sensing) or nutritional status (52, 53). Park et al. studied the *S. aureus* response to aPDI at the transcription level (54). Transcriptional profiling indicated that sublethal aPDI combined with chlorin e_6_ (Ce_6_) and laser light (λ_max_ = 664 nm) generated a bacterial stress response and activated Agr-dependent gene regulation. This result suggested the role of the Agr regulator in the *S. aureus* response to photooxidative stress generated during aPDI. Additionally, lethal aPDI conditions using two different combinations—Ce_6_ with laser light and pheophorbide a (P*a*) with red LED light (λ_max_ = 635 nm)—produced similar results. Unlike wild-type *S. aureus*, which survived the treatment conditions, the *agr* mutant strain demonstrated hypersusceptibility to aPDI. These observations proved that *S. aureus* requires an Agr regulator for protection from oxidative stress when exposed to aPDI (54). Grinholc et al. published similar conclusions (55). Clinical *S. aureus* strains lacking a functional *agr* system treated with protoporphyrin IX (PPIX) and irradiated with polarized light (620–780 nm) revealed increased susceptibility to aPDI. Moreover, the same group studied two isogenic *S. aureus* strains characterized by different a*gr* statuses (a*gr*-positive and *agr, agrA-*, a*grB-*, a*grC-*negative strains) and indicated a significant difference between a*gr*-positive and a*gr-*negative strains in response to PPIX-aPDI with 620–780 nm light (55). In contrast, Gándara et al. indicated no association between aPDI treatment using TBO with non-coherent light and *agr* gene status in *S. aureus* strains: RN6390 (*rsbU*^−^*, agr*^+^), SH1000 (*rsbU*^+^*, agr*^+^), and RN6911 (*rsbU*^−^*, agr*). However, in this case, the genetic background of the studied strains also differed with respect to another important transcription regulator, namely, *rsbU* (56). These significant variations in strain responses to aPDI may be because different PSs induce different mechanisms of photodamage.

### Sar

Another regulatory network that plays a role in the bacterial stress response is staphylococcal accessory regulator A (SarA) (57). SarA is a positive regulator of *agr* and alternative sigma factor σ^B^ activity (58, 59). It can activate the expression of various virulence factors, including enterotoxin B, toxic shock syndrome toxin (TSST-1) and most cell-bound proteins (extracellular protein A, fibrinogen and fibronectin-binding proteins). SarA downregulates several proteases, lipases and nucleases (60, 61). The involvement of SarA in photooxidative stress is not fully understood and has not been examined to date. However, Ballal and Manna published the first report revealing the role of SarA in modulating oxidative stress resistance in *S. aureus* (62). Under both aerobic and microaerophilic conditions, the levels of *sodM* and *sodA* transcripts were markedly elevated in the *sarA* mutant compared to the wild-type *S. aureus* strain. Studies of various oxidative stress-inducing chemicals indicated that in the presence of diamide, a significant increase in *sodM* transcription was observed in the isogenic *sarA* mutant strain. Additionally, a small increase in *sodA* transcription was observed in the *sarA* mutant strain in the presence of *tert*-butyl hydroperoxide (t-BOOH). However, exposure to hydrogen peroxide (H_2_O_2_) and cumene hydroperoxide (CuOOH) did not affect *sodM* and *sodA* expression. It was suggested that SarA plays a role in the regulation of Sod transcription in *S. aureus* and oxidative stress resistance (62). Research has shown, however, that the cysteine in the structure of the SarA protein that might be a redox sensing element is sensitive to alkylation rather than oxidation (63). A more in-depth analysis of the direct impact of the SarA regulatory network on the bacterial response to aPDI needs to be conducted.

### Sigma B

σ^B^ (encoded by the *sigB* gene) is an alternative sigma factor activated in the bacterial response to environmental conditions that regulates the expression of over 150 genes involved in the stress response (64, 65). The SigB operon also regulates the transcription of virulence genes, biofilm formation, membrane transport, cell internalization, persistence and antibiotic resistance (66, 67). Kossakowska-Zwierucho et al. revealed the significant role of the sigB operon of the *S. aureus* response to aPDI (68). We observed a marked decrease in the bacterial survival of sigB operon mutants compared to the wild-type *S. aureus* USA300 strain. This aPDI efficacy was noticed with the use of protoporphyrin IX diarginate (PPArg_2_), zinc phthalocyanine (ZnPC) and RB. The group obtained the same effect for other pairs of strains – RN6390 (deletion in *rsbU* gene) and SH1000 (restored deletion in *rsbU* gene) (68). Moreover, sequencing of the genes from the SigB operon (*rsbU, rsbV, rsbW, sigB*) of clinical *S. aureus* isolates highly susceptible to aPDI treatment revealed mutations in the *rsbU* gene, a positive regulator of SigB activity. Additionally, RsbU-defective *S. aureus* strains indicated a decrease in σB activity (measured by *asp23* transcript level, which is exclusively controlled by σB factor). Therefore, the SigB operon and *rsbU* gene, in particular, were proven to be essential elements for the *S. aureus* response to aPDI (68). However, similar to Kossakowska-Zwierucho et al. and Gándara et al. demonstrated that aPDI treatment was ineffective when TBO was used on RsbU-defective mutants. This ineffectiveness might be attributed to TBO being a different type of PS from PPArg_2_, ZnPC, RB. TBO is characterized as a Type I PS, acting largely via radical species, whereas the PSs involved in the study conducted by Kossakowksa-Zwierucho et al. (PPArg_2_, ZnPC, RB) are strong singlet oxygen producers (Type II PSs). It was suggested that *S. aureus* strains defective in σ^B^ are easily eradicated by aPDI treatment using photosensitizers, producing a high yield of singlet oxygen (56, 68).

## DNA Repair Enzymes

### The Role of the *recA* Gene and RecA Protein in the Phototreatment Outcome

**Figure 4:**
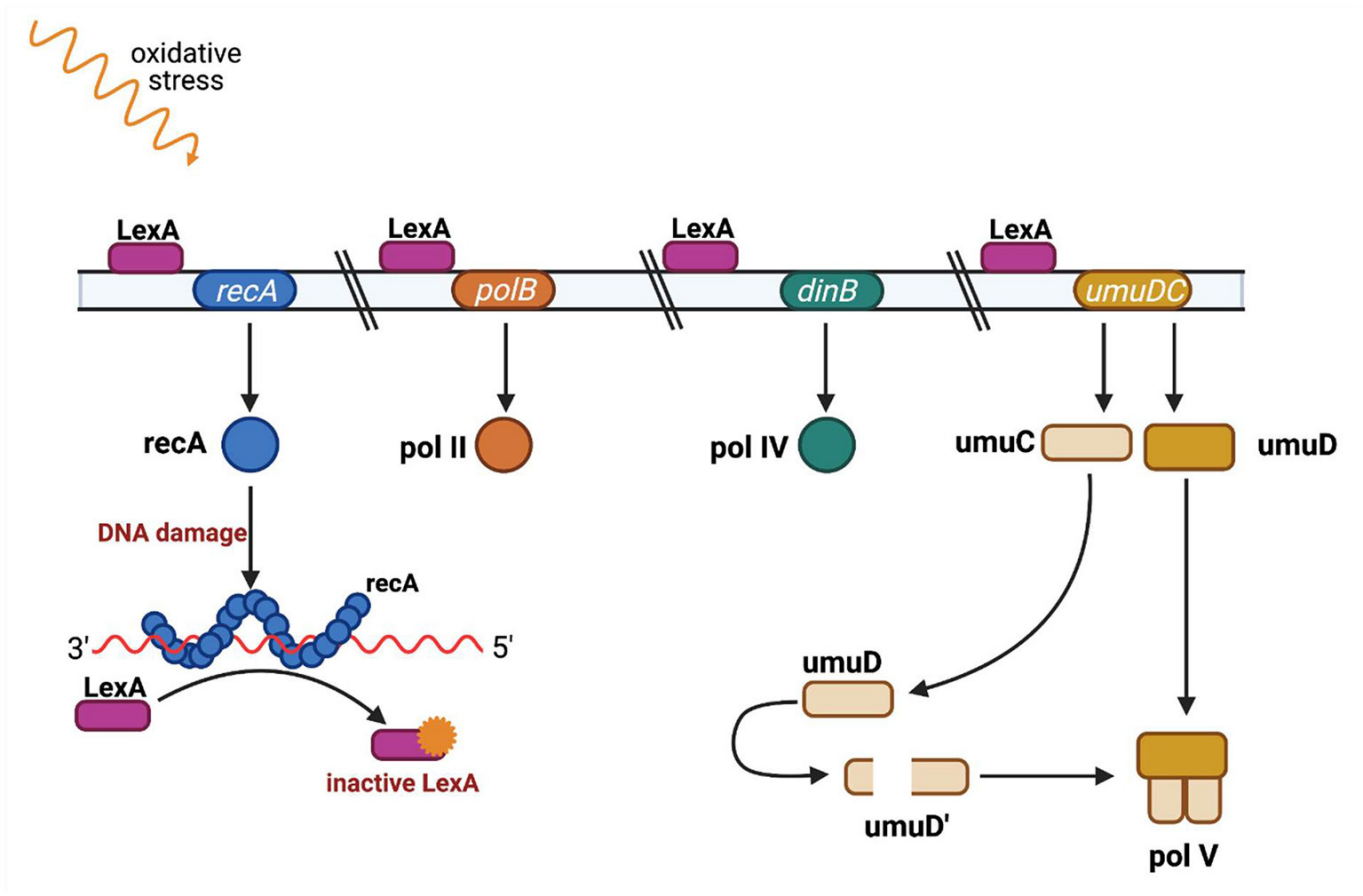
SOS regulation. The LexA protein is attached to an operator upstream of the SOS response genes and blocks their transcription. Single-stranded DNA fragments (ssDNA) accumulate when DNA is damaged (eg. as a result of UV radiation or ciprofloxacin). RecA protein binds to single-stranded DNA fragments to form nucleoprotein fibers that stimulate the SOS response. The active form of RecA protein interacts with the LexA repressor and causes LexA to dissociate from the operator. When LexA is not attached to an operator, genes responsible for DNA repair are transcribed, such as *polB* gene encoding polymerase II, *dinB* gene encoding polymerase IV, and the *umuC* and *umuD* genes encoding proteins UmuC and UmuD, which are subunits of polymerase V. Polymerases IV and V replicate damaged DNA. Adapted from Tropp (31). (Created with BioRender).

*recA* is an element of the SOS system, a bacterial response to genotoxic stress, during which bacteria induce several phenotypic changes through the differential regulation of genes and through rearranging and mutating their genome, sometimes acquiring characteristics that enable bacterial survival and adaptation to stress conditions. SOS induction is linked with the network of other essential stress responses and can be modulated under various circumstances to fit bacterial needs (69). The mechanism of the SOS response is well-known and well-described (70, 71) (Figure 4). It is induced by a single-stranded DNA (ssDNA), and two other key composites are involved in the SOS response: LexA, which is a transcriptional repressor, and RecA, which is an inducer (72). RecA binds ssDNA in the form of a nucleofilament, which is crucial for catalysis of the self-cleavage of the LexA repressor and enables expression of the SOS genes (73).

Membrane proteins and other components of the cell envelope are considered to be the major targets of oxidative stress caused by antimicrobial photoinactivation; however, the ROS produced during antimicrobial photoinactivation have the potential to cause DNA damage. Photocleavage of bacterial DNA is believed to occur as a secondary effect in extensively photoinactivated cells (6). It has been critically discussed within our recent study in which we examined the correlation between DNA damage and aPDI (74). The research investigated whether DNA damage occurs during sublethal doses of phototreatment (resulting in a 0.1-1 log_10_ unit reduction in cell viability) and demonstrated that the administration of exogenous PSs, i.e., TMPyP, RB, zinc phthalocyanine (ZnPc), NMB and TBO, or antimicrobial blue light (aBL, 405 nm), led to the excitation of endogenously produced photosensitizing chromophores, indicating that both aPDI and aBL exert (i) increased DNA damage (analyzed by gel electrophoresis), (ii) increased *recA* expression (luminescence assay conducted in the *recA-lux* strain), and (iii) increased levels of RecA protein (examined by Western blot analysis).

The same study aimed to examine whether the presence of RecA protein or inhibition of the *recA* gene could influence the effectiveness of photoinactivation; thus, wild-type USA300 JE2 and its isogenic derivative strain JE2 *recA* (*recA*-negative, NE805) were subjected to sublethal aPDI and aBL inactivation. Moreover, the wild-type USA300 JE2 strain was photoinactivated in the presence of novobiocin, is a well-known *recA*-downregulating agent, at MIC concentrations. As a result, the wild-type strain with inhibited RecA (by novobiocin) or strain NE805 with a disrupted *recA* gene demonstrated increased susceptibility to TMPyP-, RB-, ZnPc-, NMP-, and TBO-mediated aPDI as well as susceptibility to aBL. This result indicates that aPDI or aBL treatment results in DNA damage and subsequent cell death. There is also an important question of whether aPDI/aBL treatment results in increased expression of *recA*, which would indicate DNA photodamage upon aPDI or aBL. Indeed, sublethal doses of aPDI/aBL resulted in increased levels of *recA* gene expression, as measured by the increase in bioluminescent signal of *recA* promoter-*lux* constructs (75). Accordingly, RecA protein levels increased upon photoinactivation. This reveals that DNA damage occurs not only in the case of bacterial cells that are intensively photoinactivated (more than 2–3 log_10_ unit reduction), as was suggested previously (76–78), but could also be detected at lower doses of photoinactivation, when the majority of bacteria are still viable (confirmed with the cell membrane integrity assay). Within the same study, the results of the luminescence assay that enabled tracking of the activity of the *recA* promoter were subjected to Western blot analysis, demonstrating the induction of *recA* expression upon phototreatment.

In our most recent studies (20, 21) concerning aPDI and aBL, we confirmed that after consecutive sublethal phototreatments, increased *recA* expression occurs. The studies mentioned above demonstrate that aPDI/aBL treatment efficacy could be improved by controlling the DNA repair system, e.g., by inhibiting or eliminating the expression of the *recA* gene, and that *recA* is a critical element in the bacterial response to photoinactivation.

### Role of LexA in Phototreatment Outcome

LexA is a late-induced gene and can inhibit SOS induction when the genotoxic signal is stopped (LexA cleavage is no longer promoted) (69).

Our previous study examined, using a luminescence assay in *lexA*-*lux* and *recA*-*lux* strains, whether RecA determines the response of *S. aureus* to phototreatment independently of LexA (repressor of SOS regulon). The obtained results indicated that phototreatments induced both *lexA* and *recA* expression. To investigate the role of RecA alone (without LexA interference), the *S. aureus* strain with uncleavable LexA (HG001 l*exAG94E*) was used. However, the response of the mutant strain was not significantly different from that of the wild-type strain.

### Role of *the umuC* Gene in Increased Mutation Frequency and aPDI/aBL Tolerance Development

The expression of *umuCD*, like that of several other loci, is induced by DNA damage, and it is regulated by products of the *recA* and *lexA* genes (79). Similar to LexA, UmuCD production depends on proteolytic cleavage catalyzed by RecA nucleofilament (80). *umuCD* codes PolV, a stress-responsive error-prone DNA polymerase. PolV proceeds with DNA replication on damaged DNA by incorporating any base across the DNA lesion. PolV can incorporate correct or incorrect bases across the lesion on the template strand and has no proofreading activity, which may have a highly mutagenic effect due to an increased frequency of spontaneous mutation (81, 82). The outcome of such mutagenesis could lead to a phenomenon broadly defined as an adaptation (69).

Our previously published study investigated potential acceleration in the mutation frequency due to phototreatment. The rifampicin-resistant mutant selection assay is considered an adequate methodology for mutation rate estimation because, in the case of *S. aureus*, resistance to rifampicin may result from a single spontaneous mutation in *rpoB*. The group examined the development of spontaneous resistance to an antibiotic after plating on agar plates containing 4 × MIC (minimal inhibitory concentration) rifampicin to recover spontaneous antibiotic-resistant mutants. *S. aureus* USA300 JE2 did not demonstrate significant dissimilarities in response to sublethal phototreatment (0.1–1 log_10_ reduction in CFU) in comparison to basal mutation frequencies (74). We studied potential increases in the mutation frequency after the 5th, 10th, and 15th consecutive cycles of exposure to RB-mediated aPDI or aBL in the *S. aureus* USA300 JE2 strain. The results indicated that sublethal doses of aPDI or aBL induced genetic alterations in *S. aureus*. An increased mutation rate could result directly from induced DNA damage or from the activity of error-prone DNA Pol V. We employed aPDI or aBL to investigate whether phototreatments led to SOS response activation and increased expression levels of *recA* and *umuC*. In the case of both sublethal phototreatments (aPDI and aBL), we observed increased expression levels of *recA* and *umuC*. To confirm the possible SOS-dependent mechanism, we employed two transposon mutants derived from the *S. aureus* USA300 JE2 strain: with disrupted *recA* (NE805) and *umuC* gene (NE445). The obtained results indicated that aPDI, as well as aBL tolerance development, might be considered *recA-*dependent processes since no tolerance to either phototreatment upon five consecutive sublethal aPDI or aBL cycles was observed in the case of *S. aureus* NE805 lacking the functional *recA* gene. In contrast, studies revealed that only aBL tolerance acquisition is considered an *umuC*-dependent process: *S. aureus* lacking the functional *umuC* gene developed significant aPDI tolerance but expressed no aBL tolerance after five consecutive cycles of sublethal aPDI or aBL.

The described findings confirm that aPDI/aBL may result in DNA lesions that could lead to increased SOS- and *umuC*-dependent and *umuC*-independent mutagenesis.

## Heterogenous Response to APDI and Genetic Background

Our several reports indicated that susceptibility across *S. aureus* isolates to aPDI is strain dependent (55, 83–86). We examined several 100 strains in total. Under the same experimental conditions (protoporphyrin IX-mediated aPDI, wavelength range of 620–780 nm, fluence of 50 J/cm^2^), the different strains expressed a heterogeneous response to photoinactivation, ranging from 0 to 5.1 log_10_-unit reductions in viable counts.

Determination of the molecular markers underlying the mechanism of the heterogeneous bacterial response to PDI treatment would have substantial clinical importance. We performed *Alu*I and *Rsa*I digestion of the *agr* gene PCR product, which revealed existing correlations between the determined digestion profiles and PDI susceptibility (55). Furthermore, the functionality of the *agr* system affected the *S. aureus* response to PDI. Based on our results, we concluded that the *agr* gene may be a genetic factor affecting the strain-dependent response to PDI. It is also known that MRSA and MSSA have different responses to aPDI. Data obtained by our group demonstrated that MRSA strains are significantly more resistant to photoinactivation than MSSA strains. However, the difference observed did not result from the presence of *mec*, antimicrobial susceptibility or resistance mechanisms, or the ability to form biofilms *in vitro* (85). We compared the genetic backgrounds of the MRSA strains [SCC*mec* types, *spa* types and main clonal complexes, (CC)] with their susceptibility to protoporphyrin IX-mediated aPDI. SCC*mec* typing revealed no differences in response to photoinactivation. However, the detection of *spa* types and clonal complexes clustered the studied population of MRSA strains according to their response to photodynamic oxidation. Strains of CC30 (ST36) demonstrated susceptibility to photoinactivation, and CC1 manifested a decreased response to PpIX-aPDI. Moreover, *spa* typing identified isolates that were more tolerant (t032) and more susceptible to phototreatment (t051, t015) (86). The association between the response to aPDI and clonal lineages displays the important role of genetic background in aPDI effectiveness. The described results make a case for the development of a diagnostic tool with the prognostic value of aPDI efficacy according to an identified genetic background of *S. aureus* isolates; nevertheless, the mechanism underlying this phenomenon is still poorly understood.

## Physical Membrane Properties

### Lipid Composition of Bacterial Membranes

The different responses of gram-negative and gram-positive microorganisms to photoinactivation with anionic PSs are obviously due to the presence in gram-negative species (e.g., *K. pneumoniae* and *H. influenzae*) of phospholipids, lipoproteins and polysaccharides in the additional outer envelope (87). In gram-negative bacteria, the prevalence of neutral lipids is represented by phosphatidylethanolamine (PE) (70–75% of total phospholipids), and the other 25% of the lipid composition consists of anionic phosphatidylglycerols (PGs). In gram-positive bacteria, the PE content is lower. Moreover, the membranes of bacterial cells contain cardiolipin (CL), another anionic lipid. All bacterial cells contain at least 15% anionic lipids, such as CL or PG, and exposure to such anionic lipids with lipoteichoic acids (LTA) or lipopolysaccharide (LPS) reduces the diffusion of PS into the cytosol (30, 88). Differences in photoinactivation efficiency may also be related to gram-negative species having structure A in LPS and its polysaccharide composition.

Photochemical reactions that involve the type I mechanism often result in the degradation of unsaturated phospholipids due to the abstraction of allylic hydrogens from these compounds (89). The radical species thus formed may undergo reaction with oxygen to yield lipid hydroperoxides. Lipid peroxidation is detrimental to membrane integrity, leading to loss of fluidity and increased ion permeability. Other cell wall/membrane targets include aminolipids and peptides. Thus, the inactivation of membrane enzymes and receptors is also possible.

Proteins in the cell membrane are significant photoinactivation targets; thus, singlet oxygen causes protein oxidation and the formation of carbonyls and aggregates and contributes to protein folding or unfolding (90, 91). The reaction of OH• with amino acids, e.g., proline, generates protein carbonyls (92). Overall, the data presented by Dosselli et al. suggest that phototreatment leads to specific damage that depends on the localization of a PS in a cell. The presence of proteins in the cell membrane is very closely related to the dynamics of the lipid bilayer. For example, when the homeostasis of the environment is disturbed via oxidative stress conditions, the stabilization of membrane proteins by electrostatic interactions among the lipid head groups, charged amino acid residues, and fatty acids is disrupted (93). Such disturbance of membrane fluidity can lead to lethal effects. The membrane fluidity of bacterial and mammalian cells can be interrupted as a result of membrane lipid oxidation via the action of reactive oxygen species (e.g., singlet oxygen) (93). The disruption of membrane functions associated with a decrease in membrane fluidity may contribute to the bactericidal effect of photoinactivation with TBO (93). Another observation of changes in the fluidity of the cytoplasmic membrane was investigated with TBO and red light (632.8 nm) treatment of *Porphyromonas gingivalis*. To evaluate the changes in membrane fluidity, a trimethyl-ammonium diphenyl hexatriene probe (TMA-DPH) was applied. This compound contains a positively charged trimethylammonium substituent that incorporates the molecule into the plasma membrane; therefore, it binds in proportion to the free membrane surface. When *P. gingivalis* cells were treated with TBO (82 μM) and 0.88 J of helium/neon laser red light, the reduction in viable cell count was estimated as 7 log_10_. This lethal treatment and even exposure to PS alone (without irradiation) led to a significant reduction in cytoplasmic membrane fluidity. Fluorescence measurement with the TMA-DPH compound confirmed these results. The decrease in membrane fluidity after the exposure of cells to PS can be explained by the occurrence of membrane damage after exposure to visible white light. On the other hand, increased membrane fluidity is a result of TBO aggregates, which can “bind” with membrane proteins and are therefore influenced by their properties. The explanation of the increased membrane fluidity after exposure to light and TBO is not clear. The authors suggest that the obtained effect could result from peroxidation of membrane lipids or from protein-protein crosslinking (93). In another experiment, *E. coli* cells were irradiated with white light for 1.5 h in the presence of 5 μM Tri-Py+-Me-PF-tricationic porphyrin [(5,10,15-tris(1-methylpyridinium-4-yl)-20-(pentafluorophenyl)porphyrin. The application of this PS and white light led to a decrease of approximately 6 log_10_ CFU/ml. As a part of the experiment, the photodynamic oxidation of *E. coli* membrane phospholipids was examined with a lipidomic approach. The fatty acid composition and lipid hydroperoxide level in *E. coli* extracts were investigated before and after aPDI treatment. The experimental outcome revealed that upon aPDI treatment, the concentration of lipid hydroperoxides was 93.6% higher than in a non-phototreated cell. The fatty acid composition revealed that aPDI induced the formation of a high amount of lipid hydroperoxides in the *E. coli* lipid extract and decreased the unsaturated C16:1 and C18:1 fatty acids. Lipid hydroperoxide derivatives of phosphatidylethanolamines with C16:1, C18:1, and C18:2 fatty acyl chains were also detected after photoinactivation (12).

### The Role of Bacterial Pigments in aPDI

Many bacterial species, including pathogens, produce, and accumulate pigments within cell membranes. The primary function of those pigments is neutralization of oxidative stress produced by light exposure. Within microbial cells pigments additionally serve to stabilize membrane and protect lipids to assure cell envelop integrity.

### Gram-Positive Bacteria

One of the most abundant bacterial pigments produced by *S. aureus* is staphyloxanthin. The gold C30 triterpenoid [α-D-glucopyranosyl-1-O-(4,4'-diaponeurosporen-4-oate)-6-O-(12-methyltetradecanoate)] is localized in the staphylococcal membrane. This compound alternates between single and double bonds, which can quench oxidation by reactive oxygen species (94). This carotenoid compound is synthesized by six enzymes in the biosynthetic pathway encoded in the *crtOPQMN* operon and is regulated by two-component signal transduction systems (TGSs) (95). The pigmentation of *S. aureus* cells is not the main factor responsible for the resistance of this pathogen to phototreatment. This staphylococcal pigment can interact with proteins and DNA (96). The effect of *S. aureus* pigmentation on the survival rate of this pathogen was investigated with the use of chlorin-ce6 PS and red light (97). The experiment was performed on the wild-type strain *S. aureus* Newman and the non-pigmented isogenic mutant Δ*crtM* (without the gene for dehydrosqualene synthase CrtM). The application of chlorin-ce 6 at a concentration ≥128 μM led to 5 log_10_ reductions in the bacterial population of the Newman wild-type strain, and Δ*crtM* exhibited the same reduction in bacterial viability when PS was applied at concentrations ≥64 μM. These results suggest that pigmentation can influence the effectivity of photoinactivation; thus, pigmented *S. aureus* was more resistant to singlet oxygen action than its mutant without pigment (97). It was shown that the presence of carotenoids is crucial for the cell membrane integrity of *S. aureus* and influences protection from oxidative stress (94). It is also worth mentioning that polar carotenoids can modulate the fluidity properties of lipid membranes (94). Experiments performed by Kossakowska-Zwierucho et al. suggested that the enhanced membrane fluidity could be responsible for the increased effectiveness of aPDI. Membrane fluidity, which is a temperature-dependent parameter, was estimated for *S. aureus* strains (SA144, SA145, and SA147) by measuring the fluorescence polarization anisotropy (r) of 1.6-diphenyl-1.3.5-hexatriane (DPH). DPH is a lipophilic fluorescent probe that can localize in the hydrophobic regions of the cell membrane. Analysis of fluorescence anisotropy for the tested *S. aureus* strains SA144, SA145, and SA147 (which differ in the level of pigmentation) revealed that low membrane fluidity was observed for highly pigmented cells (SA144 SA147). However, for weakly pigmented cells (e.g., strain SA145), the fluorescence anisotropy value was estimated to be low, thus confirming the high level of membrane fluidity. The exposure of these three strains to photoinactivation conditions (5 nM ZnPC (zinc phthalocyanine) and red light 627 nm) revealed that strains exhibiting wild-type pigmentation and lack of pigmentation (SA 144 and SA 145) were much more sensitive to aPDI than the strain with high pigmentation (SA 147). On the other hand, the irradiation of cells with green light in the presence of RB (0.1 μM) showed a reduction in bacterial viability (by ~4 log_10_ CFU/ml), which indicates that the lack of protection against photooxidative stress was detected only for the strain lacking pigmentation (68). *Enterococcus* species are very often linked with resistance to vancomycin and human infections; however, there are many interesting species associated with soil that contain yellow pigment (98). For example, *E. casseliflavus, E. mundtii*, or *E. sulfureus* can produce carotenoids similar to those found in *S. aureus*. Experiments performed on *S. aureus* mutants deficient in the production of yellow pigment, as mentioned above, are more susceptible to photooxidative stress. For similar experimental purposes, the *E. faecalis* reference non-pigmented strain, *E. casseliflavus* (pigmented) and *Enterococcus* species environmental isolates from Avalon Beach (lightly pigmented) were used for tests. Bacteria were exposed to conditions simulating seawater at pH 8.1 and to a broad range of the visible light spectrum, 290–800 nm, without the addition of any photosensitizing agent. The results of these experiments revealed that non-pigmented *E. faecalis* strains were significantly reduced faster than pigmented strains (*E. casseliflavus* and pigmented *E. faecalis* environmental isolates) (98). The results of this research suggest that pigments of *Enterococcus spp*. could quench reactive oxygen species and be responsible for the photooxidation process.

### Gram-Negative Bacteria

Literature data on the photoinactivation and pigmentation of microorganisms were presented by Orlandi et al., who investigated the gram-negative species *P. aeruginosa*. This pathogen is known as a producer of various pigments: pyocyanin, phenazine, pyoverdine, pyorubin, and pyomelanin. These pigments are crucial in the context of photoinactivation due to their ability to survive under oxidative stress conditions. Experiments performed by Orlandi et al. aimed to identify whether *P. aeruginosa* pigments contribute to their relative tolerance to photooxidative stress. For this purpose, differently pigmented wild-type and transposon mutants of the *P. aeruginosa* PAO1 strain were used: RE- pyomelanine overproducer; BL- pyocyanin producer; and YE- pyoverdine overproducer. All four strains cultivated in LB medium or M9 with glucose were subjected to photoinactivation with TBO or TMPyP. Each photosensitizing agent has a different mechanism of action: TBO acts mainly via a type I mechanism, whereas TMPyP acts via a type II mechanism. To determine the influence of pyomelanin or pyoverdine on the oxidative stress response, mutants were treated with 25 μM TBO and irradiance at 140 J/cm^2^ or with 5 μM TMPyP and 210 J/cm^2^ of light irradiance, respectively. BL cells cultivated in M9 and glucose medium were not successfully inactivated with either PS, whereas YE, RE and wild-type BL cells were sensitive to both phototreatments (99). Other literature data showed that *P. aeruginosa* pyoverdine blue pigment can absorb visible light at 415 nm. This property suggests that this pyoverdine can participate in photodynamic reactions because it can absorb visible light (100). Colorful pigments are also found in *Prevotella intermedia* and *Prevotella nigriscens*, which are microorganisms able to colonize the oral cavity that are capable of producing black pigments. *P. gingivalis* is known as an aetiological agent of periodontal diseases. It is also easily recognized as a black pigment producer (101). Experiments performed with visible light (in the range 380 to 520 nm) revealed that the exposure of *P. intermedia* and *P. nigrescens* to visible light doses of 4.2 J/cm^2^ and of *P. melaninogenica* at a dose of 21 J/cm^2^ led to a reduction of 5 log_10_ (0.001% of survival fraction) in comparison to the control. Chromatographic analysis identified 267 ng/mg, 47 ng/mg and 41 ng/mg endogenous porphyrin in bacterial cells of *P. intermedia, P. nigrescens*, and *P. melaninogenica*, respectively (102). Another study also confirmed photoinactivation with aBL against species producing the black iron pigment (103). The pigment produced by *P. gingivalis, P. intermedia* and *P. nigriscens* is reported in the literature as μ-oxo bishaem ([Fe(III)PPIX]_2_O) (104). A bacterial cell-surface layer of μ-oxo bishaem can act defensively by protecting bacterial cells against hydrogen peroxide (104). The abovementioned species use haem as an iron source for their growth; thus, they can accumulate black pigment that mainly consists of μ-oxobishaem at the cell surface. Despite the predomination of iron-protoporphyrin pigment, these species can also accumulate iron-free PpIX, which can be easily excited with visible light. This process leads to energy transfer from the porphyrin triplet state to molecular oxygen to produce singlet oxygen (type II mechanism of action) (102, 103). The effectiveness of photoinactivation with the engagement of free PpIX has been proven many times in the literature, and the use of exogenously administered PSs, e.g., TBO, highlights another interesting fact related to the response of cells to photoinactivation treatment (93, 101).

## Photosensitizer Accumulation in Cells

**Figure 5:**
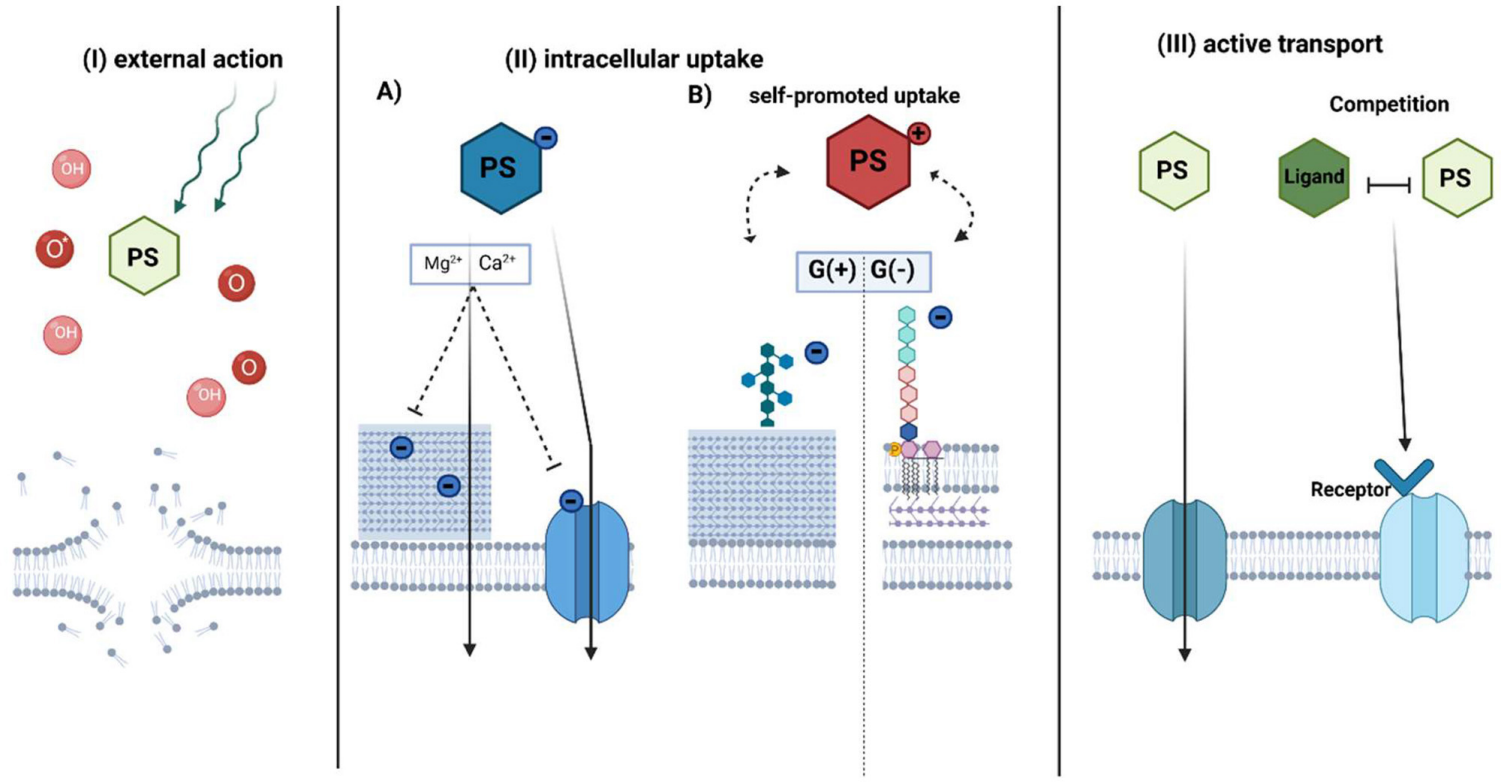
Proposed mechanisms of photosensitizer accumulation in aPDI treatment. The photosensitizer uptake mechanism can be divided into three general pathways: (I) external action, (II) intracellular action, and III) active transport-dependent accumulation. In the external action **(I)**, PS does not accumulate inside the bacterial cell, however, due to the diffusion length of generated ROS (OH) or singlet oxygen (O^*^) upon irradiation, it causes the membrane disruption. The intracellular action **(II)** depends on the PS charge. (A) Anionic PS is accumulated by electrostatic interactions or active transport. The addition of divalent cations neutralizes negative charge on the bacterial envelope and promotes uptake of negatively charged PS through membranes. (B) Cationic PSs use mechanism known as “self-promoted uptake.” The positively charged compound is bound to the negatively charged teichoic acids on the peptidoglycan of the G(+) or lipopolysaccharides on the outer membrane of G(-) bacteria. The photosensitizer is associated with the surface layer, while after photosensitization it promotes cell disruption. In active transport **(III)**, PS is taken up inward through a membrane protein channel such as porins or by mimicking receptor binding due to structure similarity to its natural ligand. PS-photosensitizer, O^*^- singlet oxygen, OH- radicals referred in general as ROS. (Created with BioRender).

The accumulation of a PS is one of the crucial factors to establish an efficient photodynamic approach for bacterial eradication. However, according to the literature data, this intracellular accumulation is not a necessary element for aPDI. Accumulation in cells is generally described by three main mechanisms: (I) external action, (II) intracellular action (including self-promoted uptake), and (III) active transport (Figure 5). All of them have provided efficient photokilling of both gram-positive and gram-negative bacteria (6). However, there are additional factors that affect the accumulation of PSs.

### (I) External Action

Photodynamic targets (such as cellular components and nucleic acids) are dependent on the localization of PS and the maximal diffusion length of generated ROS and singlet oxygen (105)_._ There is no requirement for a PS to be intracellularly taken up to promote oxidative damage; thus, the proximity of PS to bacteria might promote efficient photokilling. According to recent studies, cationic charged TMPyP does not accumulate inside the cells of *E. coli*. While remaining outside, however, it could provide significant phototoxicity by singlet oxygen generation (106). Additionally, Gollmer et al. showed that TMPyP accumulates minimally in the cytoplasm and the cell wall of *S. aureus* but only after cell wall damage (107). Nevertheless, these results are not in accordance with previous studies on the localization of TMPyP (108), so further investigation of the external action of PSs is important.

### (II) Intracellular Uptake

Simple diffusion of PSs through physical barriers, such as membranes, is restricted to either gram-positive or gram-negative bacteria due to the cell wall structure (109). The primary issue for uptake is the structural organization and charge of the PS. Anionic PSs accumulate in bacterial cells by electrostatic interactions or via active transport (110). The addition of divalent ions such as Mg^2+^ or Ca^2+^ promotes the accumulation of such PSs through higher activation of protein transporters or neutralization of the negative charges of bacterial envelopes, thus reducing the repulsion between anionic PS and the bacterial cell wall (110). In general, gram-negative microorganisms are more aPDI-resistant with applied anionic or neutral PS than gram-positive bacteria (111). Only by adding membrane-disrupting agents such as EDTA (112) or polycationic peptide, for example, polymixin B (113–115), can these PSs accumulate in gram-negative cells (110).

Cationic PSs could be intracellularly accumulated by electrostatic bonds in a self-promoted uptake mechanism (110). The peptidoglycans of gram-positive bacteria include negatively charged teichoic acids that constitute binding sites for PSs. Cationic PSs such as MB or TBO could also be taken up into gram-negative cells by a self-promoted mechanism. MB binds as a dimer to negatively charged LPS, causing an association with the surface layer and cell disruption after photosensitization (116). Negatively charged lipopolysaccharides are stabilized by electrostatic bonds with surrounding divalent ions such as Mg^2+^ or Ca^2+^, which act compatibly with PS. Divalent ions promote the inhibitory effect on cationic PS uptake. Saji et al. showed that the accumulation of MB by a self-promoted mechanism was reflected by the effectiveness of aPDI. A reduction of *E. faecalis* cells was achieved with 6.25 μM MB in the absence of divalent ions upon irradiation. In the presence of divalent ions, a PS concentration of up to 100 μM was achieved. Additive divalent ions inhibit MB binding to LPS (117) and accumulation, which was also confirmed in uptake assays (110).

Compared to MB, the cationic PS TBO, displayed a higher reduction in bacterial burden after photodynamic treatment (118, 119). Spectrophotometric studies revealed that TBO has a greater affinity for different types of LPS from MB (116). Contrary to expectations, MB-mediated photoinactivation was significantly more inhibited by CaCl_2_ than TBO-mediated photoinactivation. It was suggested that MB could also be electrostatically bound to charged amino acids in proteins such as siderophores (120). The complex of MB and the protein receptor on siderophores translocates PS through the membrane. However, the mechanism involving LPS is much more efficient in photokilling. The two mechanisms could coexist and influence aPDI effectiveness (120).

The self-promoted uptake pathway was also considered a mechanism of action for cationic pyridinium zinc phthalocyanine in *E. coli* (121) or cationic *meso-*substituted porphyrins in *P. aeruginosa* (122), *Vibro anguillarum* and *E. coli* cells (123). However, the number of cationic charges associated with the neutral PS might influence its hydrophobicity (124, 125). The increased amphiphilic character of porphyrins seems to enhance their affinity to the envelope of gram-negative bacteria, which improves accumulation. This might be accomplished by the asymmetric charge distribution or by adding cationic charges (6). Different numbers of positive charges of meso-porphyrins result in changes in the log_10_ reduction of aPDI (125–127).

### (III) Active Transport

In active transport, PSs might be bound to the bacterial cell wall and then transported into the plasma membrane. In general, hydrophilic PSs are better able to penetrate through protein channels such as porins. Porins transport low molecular weight (up to 700 kDa) hydrophilic compounds (128, 129) and could mediate the active transport of PS. Divalent ions and trypsin may influence the uptake of PSs (110, 122). Trypsin promotes the functional impairment of cell wall-associated proteins, while divalent ions might have an impact on protein activity. The mechanism of RB uptake was proposed to be mediated by proteins based on the inhibitory aPDI effect after treatment of cells with trypsin (110).

The mechanism of exogenous porphyrin accumulation is still not fully understood. One of the proposed hypotheses is recognition by the haem transport machinery due to its similar structure to Fe^3+^*-*protoporphyrin IX (known as a heme). The well-characterized transport system is the staphylococcal iron-regulated surface determinant system (Isd), described in detail in many research studies by the Skaar group (130–133). Briefly, in a low-iron environment, heme-acquisition machinery recognizes and binds free haem or haemoproteins using an Isd protein system (130, 133). Haem is transferred to the cytoplasm using ABC-like membrane transporters, then degraded by an oxygenase to release free iron from the porphyrin structure (134). Paradoxically, too high intracellular concentration could be a challenge for *S. aureus* due to the toxicity of free iron (131). The HssRS (two-component system required for haem sensing)/HrtAB (haem-regulated transporter) system senses and pumps out an excess haem (135). Based on our investigation, mutants with impaired HrtA protein function accumulated PpIX in the largest amount and were the most efficiently eradicated upon PpIX-based photoinactivation in comparison to other haem transporter mutants, ΔIsdD, ΔHtsA, and wild-type *S. aureus*. However, the observed results do not correlate solely to protein function but also to a physical membrane modification that lacks the HrtA protein. It cannot be ruled out that PpIX may be a substrate for the HrtAB efflux pump (136). Metalloporphyrins, haem analogs, could be a potential group for targeting haem machinery (137). Metals in the oxidation state (III) have an affinity to the haem receptor on the Isd protein and mimic the properties of the natural ligand (138). Studies on the accumulation of metalloporphyrins showed that *S. aureus* cannot detoxify most of the toxic analogs through pump efflux in the same manner as heme. Most of them accumulate in the staphylococcal cell membrane (135). Recent studies of aPDI with Ga^3+^ PPIX showed rapid, diffusion-limited uptake of PS correlated with the appearance of cell-surface hemin receptors. Additionally, photoinactivation was more potent than in the case of PpIX (139). These indications might open a novel pathway for metalloporphyrins in the aPDI approach.

Microbial efflux pumps (MEPs) are transport proteins localized in the bacterial membrane that are involved in the detoxification process of toxic substances in bacteria. Some efflux pumps selectively pump out specific antibiotics, while multidrug resistance pumps (MDRs) remove structurally diverse compounds with a different mechanism of action. A well-known major facilitator-type MEP is NorA in *S. aureus*, while in gram-negative bacteria, a three-component RND (resistance nodulation division) efflux pump is typified by MexAB-OprM in *P. aeruginosa*. It has been suggested that amphipathic cations are natural substrates of MEP. Tegos et al. published studies on the accumulation of cationic phenothiazinium salts in knock-out and overexpressed MDR pump mutants of *S. aureus, P. aeruginosa*, and *E. coli*. The MDR-aberrant phenotype indicated an increased accumulation of all phenothiazinium dyes in each MDR knockout mutant in the studied species. The uptake of non-phenothiazinium dyes remained unchanged in NorA(+), NorA(–), and wild-type *S. aureus*, which indicated that these compounds are not recognized by MDRs (140). Adding inhibitors specific to the type of efflux pump promoted the cellular uptake of phenothiazinium salts in *S. aureus* and *P. aeruginosa* and was correlated with a higher aPDI efficiency (141). NorA inhibitors covalently attached to the structure of MB demonstrated the efficiency of aPDI and higher accumulation in MRSA (methicillin-resistant *S. aureus*) than MB alone (142). Additionally, those hybrids showed enhanced *E. coli* and *Acinetobacter baumannii* cell killing upon irradiation under both *in vitro* and *in vivo* conditions, despite neither species expressing this type of efflux pump (143). In contrast, Grinholc et al. observed that amphiphilic cationic PPArg_2_ was not a substrate for the NorA efflux pump even when an efflux inhibitor was used (84). Efflux pumps could be potential targets for increasing the intracellular level of suitable PSs and improving the activity of aPDI toward gram-positive and gram-negative bacteria.

### (IV) Additive Factors

Photosensitizer affinity to the bacterial cell depends on more than the cell structure or physicochemical properties (Figure 1). Factors such as the composition of the culture medium and its additive components, PS concentration, solvent or cell density might also have a significant impact on the effectiveness of accumulation.

As referred previously, monovalent and divalent ions had a concentration-dependent effect on PS accumulation in gram-positive and gram-negative bacteria (122). Divalent cations such as Ca^2+^ have a stronger effect on ionic strength than monovalent cations such as Na^+^. The ionic strength of a solution has a strong impact on the activity of polyvalent ions (114, 122). Additionally, the presence of EDTA might exhibit increased or decreased uptake, depending on the charge of the PS, which is also reflected in aPDI efficiency (110). The protein composition in the medium also influences the effectiveness of accumulation. A 7-fold reduction in the protein content in the medium resulted in a greater reduction in A. *baumannii* cultures after photoinactivation with TMPyP (144). For instance, proteins such as human albumin or lipoproteins interact with PPIX (136). However, different proteins have limited affinity for certain PSs (144).

Another approach for a higher uptake of PS is preincubation with antimicrobial peptides (AMPs). AMPs are amphipathic amino acids with up to 50 amino acids. Positively charged peptides are bound to anionic groups on the bacterial membrane and promote the creation of pores (145). A study by our group revealed a 20-fold decrease in RB concentration and fluence in the photoinactivation of *P. aeruginosa* after supplementation with AMPs. Combined treatments with RB and PEX (pexiganan) or CAM (camel) resulted in a doubled uptake rate of RB in comparison to RB alone (146). The uptake of MB in the presence of AMP aurein 1.2 was also double rated. However, studies on chlorin-e6 combined with AMPs showed a decrease in uptake of PS with a synergistic effect in aPDI. This synergy was observed only in photokilling with MB and chlorin-e6, which indicated PS-dependent action (147).

Effective uptake also relies on the PS concentration. Cationic PSs are more efficient for aPDI and accumulate at a lower concentration than neutral or anionic PSs. The uptake of all types of PS was inversely dependent on cell density (13). Notably, the solvent might also contribute to the accumulation process, resulting in different aPDI effects. Studies on MB uptake in *E. faecalis* revealed higher accumulation in water, while photokilling was reduced in comparison to other solvents (148). Water as a solvent promotes the aggregation of MB and TBO at the cell wall of bacteria (119). Aggregated PS exhibits a reduction in the quantum yield of singlet oxygen, resulting in diminished bacterial eradication upon light illumination (76). The most appropriate solvent for PS should be taken into consideration while optimizing the aPDI approach.

The uptake rate of PS may be strain dependent. In the work of both Gad et al. and Tegos et al., there was a significant difference in uptake and aPDI efficiency in various *S. aureus* strains; however, there was no relevant correlation (149). Investigations by our team suggested that the level of PPArg_2_ uptake was linked to the level of aPDI susceptibility of MRSA and MSSA (84, 150). Interestingly, aPDI tolerance might be a result of the decreased accumulation of PS. Recent studies by Pierański et al. showed decreased accumulation of RB inside an aPDI-tolerant *S. agalactiae* strain after 10 cycles of aPDI treatment. Without selective pressure, uptake remained at the same level. Multiple aPDI treatments promote cell envelope-related modifications, resulting in decreased PS uptake. Lower PS availability results in less effective photokilling of bacteria. This study is the first investigation of decreased PS uptake as a possible microbial strategy to gain aPDI tolerance (21).

## Conclusions

In this review, we discuss several issues contributing to the various responses of bacteria to aPDI or aBL. All the issues addressed contribute to the aPDI or aBL outcome to a greater or lesser extent. The mechanisms of aPDI or aBL are non-specific; the toxic effects of ROS are directed at many cellular targets. Thus, various bacteria for photoinactivation depend on a particular photosensitizer that accumulates in the cell to produce photoinduced ROS, which ultimately causes cytotoxicity and cell death. The potential problem with aPDI or aBL lies in the potential to introduce damage to DNA, which, in the case of SOS induction, may lead to the mutation and fixation of a particular genetic feature in the population that equips the bacteria better to resist aPDI or aBL. Another very important aspect of aPDI should be taken into account, namely photoinactivation based on photosensitizing compounds bound to a solid support. The direction of research on solid-based photosensitizing compounds focuses primarily on the production of materials with specific properties: efficient ROS generation, resistance to self-quenching, high photooxidation efficiency. These properties may differ for the same photosensitizing compound depending on whether it will be free or bound to a solid material (151).

For solid-based PS, two aspects must be considered: (i) the first situation where the photosensitizing compound cannot enter the bacterial cell due to its immobilization on an insoluble carrier. In this situation, the cell is affected through the extracellular structures associated with the cell wall and the cell membrane. In this case, the signal generated by aPDI is strong enough to cause the desired phototoxic effect and cell death. Potentially, one could also consider the situation (ii), in which the signal generated at the level of the external cell structures is sent inside the cell and cytotoxic effects are induced inside the cell. However, this is a purely hypothetical situation, and so far no experimental data has shown such a situation. In the light of the current state of knowledge, the action of photosensitizing compounds related to the solid medium consists in destroying external cell structures, especially the cytoplasmic membrane (gram-positive bacteria) and the cytoplasmic and outer membrane (gram-negative bacteria) that lead to leakage of cellular content or disruption of critical enzymes (152). This type of in-depth analysis to explain the mechanism underlying the effects of aPDI or aBL on specific photosensitizers either in solution or based on solid material requires further study.

## Author Contributions

AR-Z wrote a fragment related to the influence of aPDI on DNA. AW prepared a fragment on the properties of cell membranes. KM prepared a fragment on accumulation. MP described oxidative stress. PO described stress cell regulators. MG prepared an introduction and edited manuscript. JN did literature search, prepared an outline of the manuscript, edited the entire text, made a substantive, stylistic, and linguistic correction. All authors contributed to the article and approved the submitted version.

## Conflict of Interest

The authors declare that the research was conducted in the absence of any commercial or financial relationships that could be construed as a potential conflict of interest.
